# Investigation of the cellular and molecular effects of dehydrozingerone formulation on various days of diabetic wound repair

**DOI:** 10.1007/s13205-024-03963-2

**Published:** 2024-04-01

**Authors:** Farmiza Begum, Krishnadas Nandakumar, Rekha Raghuveer Shenoy

**Affiliations:** 1 Department of Pharmacology, Manipal College of Pharmaceutical Sciences, Manipal Academy of Higher Education, Manipal, Karnataka 576104 India; 2 Department of Pharmacology, Vaagdevi Pharmacy College, Bollikunta, Warangal, Telangana 506005 India

**Keywords:** Diabetes, Excisional wound model, TNF-α, CD31 angiogenesis, MAPK signaling

## Abstract

Cases of diabetes are significantly increasing year by year, attracting the attention of medical professionals and researchers to focus on diabetes and its underlying complications. One among such are diabetic wounds which are difficult to heal, creating severe implications in the day-to-day chores of not only patients, but also family members. Dehydrozingerone (DHZ) is known to possess various effects like anti-inflammatory, anti-microbial, antioxidant, and wound-healing properties. The effect of DHZ on different phases of diabetic wound healing remains untested. Hence, this study was proposed to find out the effect of oral and topical formulation of DHZ on day 5, 10 and 15 of diabetic wound healing. Excisional wounds were created on the dorsal side of animals using punch biopsy to mimic human diabetic wounds. Topical DHZ gel (100 mg in 1 gm of gel) was prepared using 1% Carbopol 934 and was applied twice a day. The treated groups had increased percentage of wound closure; western blotting suggested that DHZ significantly increased ERK and JNK levels and decreased TNF and MMP 2 and 9 levels. From histopathological studies, it was observed that angiogenesis, collagen formation, granulation tissue formation, and fibroblast proliferation were improved on days 5, 10, and 15 of diabetic wound healing. These findings indicate that DHZ (both systemic and topical) are effective during the early phases of wound healing which gets impaired in diabetic wounds. Dehydrozingerone accelerated diabetic wound healing by regulating the various hallmarks of wound healing process.

## Introduction

Diabetes mellitus is a multifaceted metabolic disease which affects nearly 340 million people globally (Gooyit et al. 2014; Yazdanpanah 2015). People with diabetes have impaired ability in metabolizing glucose resulting in hyperglycemia with many complications, including damage to blood vessels and delayed healing of wounds. In diabetics, damage to the blood vessels leads to hypoxia, which is the contributing factor of delayed wounds, initiating inflammation and triggering release of reactive oxygen species (ROS), which thwarts wound closure by degrading the extracellular matrix. Every year, tens of thousands of people with diabetes have a lower limb amputated, because a small foot wound failed to heal (Mustoe 2004; Alleva et al. 2005). With diabetes rates on the rise, scientists are eagerly searching for effective treatments for the ulcers that develop from these unhealed wounds.

Normal healing process involves three programmed phases, viz., inflammation, proliferation, and remodeling, which overlap and are continuous with one another. In diabetes, these phases are disorganized, due to hypoxia, decreased growth factor production, compromised angiogenesis, imbalanced cell infiltration, and dysfunctional macrophages (Okonkwo and Dipietro 2017; Burgess et al. 2021). Diabetic patients with wounds are refractory to present therapeutics, and hence wounds, instead of healing, persist for months and years failing to heal. It is very important to develop a therapeutic strategy which is also economical in terms of usage and can act on all the phases of healing (Spampinato et al. 2020).

With a rising number of diabetics globally, diabetic wounds are a clinically significant complication of diabetes. India and China have one of the largest populations of people with diabetes, placing a tremendous burden on the fragile healthcare system of developing countries. The sociological factors of diet, along with a robust and booming economy, have supported further development of lifestyle disorders in the country. A rising number of diabetics would mean increasing complications and, thus, a burden on the nation. Diabetic foot is a complication that involves chronic wounds that resist healing and frequently lead to the amputation of the limb, leaving the patient disabled and with a poor quality of life. In a nation like India, this leads to the possibility of the patient losing their job, in turn causing the patient's family to bear a tremendous financial burden, along with the patient having to be admitted to the hospital more frequently. This can lead to a cycle of hospitalization that continues until the patient passes away. In India, 20% of diabetics who develop diabetic foot ulcers would ultimately need to have their feet amputated (Ghosh and Valia 2017; Keni et al. 2022). The total incidence of ulcers in diabetics can vary between 19 and 34% (Armstrong et al. 2017). Hence, it is not shocking that a maximum proportion undergoes amputations, thus decreasing the patient’s quality of life and demanding costly treatments. An estimated diabetic wound market ranges between 7 billion USD in the present day, which can increase to a whopping 11 billion USD by 2027, thus making it crucial for the development of new effective therapeutic strategies against this disease condition (Glover et al. 2021; Burgess et al. 2021).

As of now, a limited number of FDA-approved treatments and devices are currently available, namely becaplermin (Nagai and Embil 2002) which is a PDGF-BB growth factor, collagenase clostridial ointments, cell-based therapy, shock wave therapy, and omnigraft (Ramirez-Acuña et al. 2019). These options focus on symptomatic treatment and either show less efficacy and side effects, or have short half-life. The mentioned drug, becaplermin, on long-term usage, leads to carcinogenicity (Nagai and Embil 2002). Hence, there is no proper standard drug which can heal diabetic wounds. The present treatment options should focus on lesser side effects, promising efficacy, and prolonged half-life of the compounds.

Dehydrozingerone is known to exert various activities such as anti-inflammatory, anti-microbial, antioxidant, and wound healing (Rajakumar and Rao 1994; Rao et al. 2011; Burmudžija et al. 2017). The wound-healing activity of DHZ has been reported in normal wounds (Rao et al. 2011), but its mechanism and activity in high-fat diet-induced diabetic wounds have not been established yet. The results from the previous articles (Hayun et al. 2018; Rao et al. 2011Yogosawa et al. 2012) suggest that it might be a promising antioxidant for developing novel compounds, which can heal delayed wounds. Thus, the aim of the present study was to evaluate the effect of systemic and topical dehydrozingerone (DHZ) on various days of diabetic wound healing.

## Methods

### Experimental animals

Male healthy Wistar rats of 4 weeks of age were procured for the study and maintained according to the Committee for the Purpose of Control and Supervision of Experiments on Animals (CPCSEA) guidelines. The rats were fed a regular animal pellet diet: a high-fat diet and water ad libitum. They were housed at standard housing conditions of temperature (23 °C ± 12 °C), humidity (45 ± 5%), and 12 h light and dark cycle. The animals were kept in polypropylene cages, and all procedures on them were carried out in an aseptic environment. The study protocol was verified by the Institutional Animal Ethics Committee. (IAEC/KMC/18/2019).

### Induction of diabetes

The animals were divided into four groups each. The group I- control and group II- disease control were fed a high-fat diet for 3 months, followed by 35 mg/kg of streptozotocin i.p.*,* later stabilized for one  month. Group III was given dehydrozingerone (DHZ) 100 mg/kg orally, and group IV animals were applied topical DHZ gel formulation (Ozturk 2016; Gourishetti et al. 2020; Keni et al. 2020). Blood glucose levels were checked using glucometer (Contour plus glucometer, Ascensia diabetes products). The animals with blood glucose levels above 300 mg/dl were selected for the study.

### Synthesis of DHZ

2.5 grams of vanillin in 10 ml of acetone was dissolved in 10 ml of cold water. 1.4 g of sodium hydroxide was dissolved in a separate beaker and poured slowly to the above solution. At 300 rpm, stirring was done for 1.5 h using a magnetic stirrer till a bright yellow precipitate was obtained. The mixture was kept overnight for evaporation of acetone at room temperature, so that the precipitate turned blood red. Into a 500 ml beaker, the blood red precipitate was transferred by adding 300 ml of cold water along with 100 g of crushed ice. The beaker was kept in an ice bath and then the solution was acidified with 10% of HCl, till a pH of 6 was attained, giving a turmeric yellow precipitate at the bottom of the beaker. Using vacuum pump, the solution was filtered and dried in open air for 2 h. The practical yield was calculated. The product was recrystallized using 40% ethanol. Shiny yellow crystals were obtained and their melting point was determined (Hayun et al. 2018). Spectral analysis (IR, NMR, mass spectroscopy) of drug was performed at Divis Laboratories, Hyderabad.

### Solubility profile

Dehydrozingerone was added to 2 ml of water and kept in a Roto-spin (100 rpm) for about 48 h at 37 °C to determine the maximum aqueous solubility of dehydrozingerone. The solution was centrifuged for 10 min at 10,000 rpm and then filtered using 0.45 Whatman filter. The quantity of drug solubilized in each buffer was measured by a UV–visible spectrophotometer and quantity determined using the regression equation derived from the calibration curve (Tomar and Gupta 2015; Aiyalu et al. 2016).

### Topical gel formulation

Carbopol 934 (1%) was added to 10 ml of water and stirred on a magnetic stirrer for 2 h, followed by addition of propylene glycol (q.s.) and triethanolamine (q.s) for neutralizing the base. Stirring was continued till a transparent gel appeared. 100 mg of DHZ was added to 1 g of gel base and stirring was continued till a homogenized mixture was obtained (Tomar and Gupta 2015; Aiyalu et al. 2016).

### Determination of the pH of the gel

The pH was determined using the Eutech pH-510. Before utilizing the pH meter, standard buffers of pH 4 and 7 were used for the calibration (Tomar and Gupta 2015).

### Determination of spreadability

A circle was drawn on the center of one of the Petri dishes, 0.5 g of the sample was placed in the circle of the glass plate, and another Petri dish was placed over it. Over the Petri plates, a known weight was kept. Then the spreading area to the mass applied was estimated (Tomar and Gupta 2015).

### Determination of viscosity

Viscosity was measured using a **viscometer** (Aiyalu et al. 2016).

### Determination of the content of the drug

About 1.0 g of the gel was collected and solubilized in a volumetric flask of 10 ml ethanol using a bath sonicator and the samples were then filtered using Whatman filter paper. 1 mL of the filtered solution was diluted using methanol before being examined by a UV–visible spectrophotometer. The drug contents were determined using the plot equation for phosphate buffer pH 7.4 and 6.8 (Tomar and Gupta 2015; Aiyalu et al. 2016).

### In vitro permeation studies

A day prior to diffusion experiments, Himedia membrane was immersed in the phosphate buffer of pH 5.6 and 7.4 and methanol (2% w/v). A precise content of the gel 1.0 g was added to the membrane, which was placed on the Franz diffusion cell. The whole membrane was in contact with the phosphate buffer of pH 5.6 and 7.4 present in the receptor compartment as the diffusion medium, respectively. A magnetic bead was utilized to agitate the fluid in the receptor compartment. The entire setup was placed on the magnetic stirrer, and the buffer was constantly swirled at 100 rpm while the temperature was maintained at 37 ± 1 °C. At regular intervals, 2 ml of the sample was extracted and replaced with the same. The samples were analyzed for the amount released (Tomar and Gupta 2015; Aiyalu et al. 2016).

### Drug release kinetic studies

The kinetics of drug release of the improved dosage forms were investigated with grafting ex vivo permeation studies in several kinetic models such as zero order and first order and other mechanisms like Higuchi and Korsmeyer–Peppas. The drug release kinetic models were carried out using an optimized co-amorphous formulation (Tomar and Gupta 2015; Aiyalu et al. 2016; Wójcik-Pastuszka et al. 2019).

### Excisional wound model

Circular wounds of about 1 cm were made on the dorsal region of rats' skin using a punch biopsy and observed on 5, 10, and 15 days. The control and disease groups were given saline orally and for the treatment groups 100 mg/kg of dehydrozingerone was administered via the oral route in one group and DHZ gel twice a day was applied to the other group (Shenoy et al. 2011; Rao et al. 2011).

### Measurement of wound contraction

On the 5th, 10th, and 15th days of treatment, the wounds were photographed, and the wound size was measured using Image J software. The percentage of wound closure was determined using the formula (Rao et al. 2011):

$${\text{Percentage}}\;{\text{Closure}}\;{\text{of}}\;{\text{wound }} = \frac{{{\text{area}}\;{\text{of}}\;{\text{wound}}\;{\text{on}}\;{\text{day}}\;{\text{zero}} - {\text{area}}\;{\text{of}}\;{\text{wound}}\;{\text{on}}\;{\text{respective}}\;{\text{days}}}}{{{\text{Area}}\;{\text{of}}\;{\text{the}}\;{\text{wound}}\;{\text{on}}\;{\text{day}}\;{\text{zero}}\;}} \times 100$$

### Expression of markers involved on various days of wound healing using western blotting

Homogenization of wound tissue samples using POLYTRON-800 homogenizer was done, using RIPA buffer (radio immunoprecipitation assay) for lysis of cells with protease inhibitor and phosphatase inhibitor. The obtained lysate was centrifuged at 16,000 rpm for 20 min, the supernatant was collected, and protein levels were estimated. 50 ug protein was separated using SDS-PAGE (10%) electrophoresis, then transferred onto PVDF (polyvinylidene difluoride) membrane. The membrane was blocked using 3% BSA in 1X TBST for two hours. The membrane was washed three times, 10 min each, using TBST, then incubated with the primary antibodies ERK, p-ERK, JNK, p-JNK, AMPK, pAMPK, VEGF, MMP-2, MMP-9, TNF-α (1:1000) and COL-1 (1:500) at 4 °C overnight, followed by incubation with horseradish peroxidase-conjugated anti-IgG Secondary antibody (1:10,000) for 2 h. The blots were detected using ECL solution (Westar Antares, Cyanagen, Bologna, Italy) in Syngene GBox Chemi XRQ gel documentation system. Quantification of protein band intensity was done using ImageJ software and relative density was calculated in comparison to alpha-tubulin expression (Begum et al. 2023).

### Immunohistochemistry (CD31 angiogenesis)

Formation of new blood vessels is crucial in the healing of wounds (Dipietro et al. 2016), which facilitates debris removal and helps in the development of granulation tissue that assists in the closure of wound. CD31 is found on endothelial cell surface and is a major marker for angiogenesis (DeLisser et al. 1997). The slides were entirely submerged in a retrieval solution of pre-heated antigen and boiled for about 15 min. These slides were cooled at room temperature in the antigen retrieval solution for 20 min. Using 1X PBS, the slides were washed 5 min on a shaker with mild agitation. Blocking was done for 1 h at room temperature. It was then washed using 1X PBS three times, 5 min each. The blocking solution was diluted with CD-31 Antibody accordingly, and the primary antibody solution was added to the tissue and covered. Incubation was done overnight at 4 °C. Tissue slides were washed three times using 1X PBS for 5 min each, followed by incubation using HRP-labeled secondary antibody for 1 h at room temperature. The slides were washed again with 1X PBS three to five times (5 min each wash). The peroxidase activity was developed using diaminobenzidine tetrahydrochloride (DAB) at room temperature for 5 min. The sections were washed and observed under a microscope. The brown-colored stained regions were then counted using Image J (Keni et al. 2023).

### Histopathological assessment

Excision wound tissue was stored in 10% formalin and fixed in paraffin following the standard laboratory procedure for histopathology analysis. 5 µm-thick sections of paraffin-fixed tissue were achieved and staining was done using hematoxylin and eosin and Masson trichrome. Slides were examined under light microscope (Begum et al. 2023).

### Statistical analysis

All the results were expressed as mean ± SEM and evaluated using one-way and two-way ANOVA using GraphPad Prism software version 8.4.2.

## Results

**Fig. 1:**
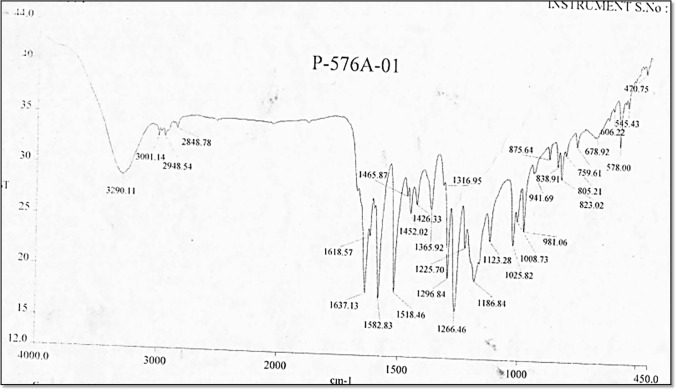
FT-IR of synthesized dehydrozingerone. The characteristic and diagnostic bands were visible at regions 3290–1582 cm^−1^ which confirms the product to be DHZ

**Fig. 2:**
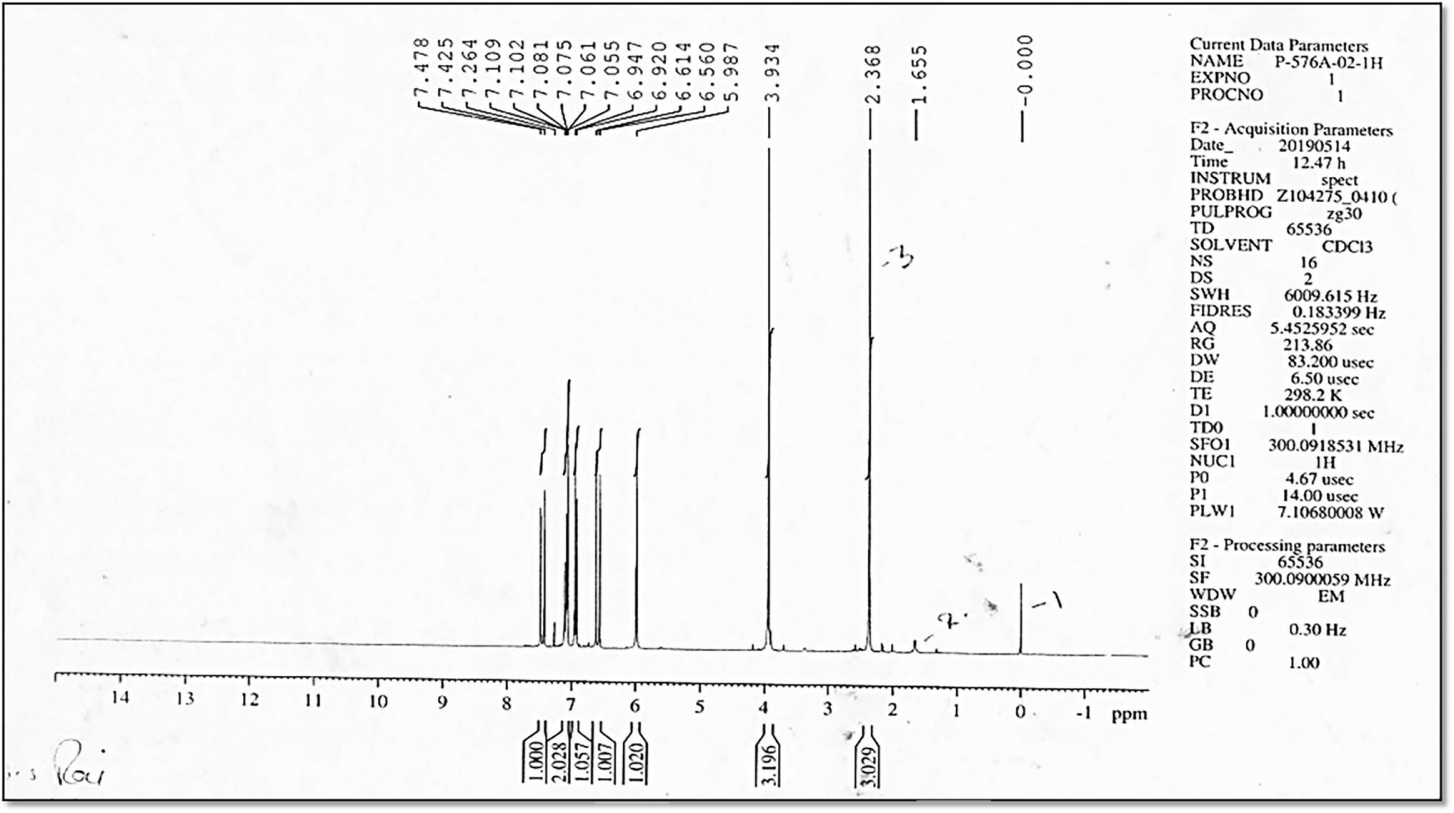
^1^H-NMR of synthesized dehydrozingerone

**Fig. 3:**
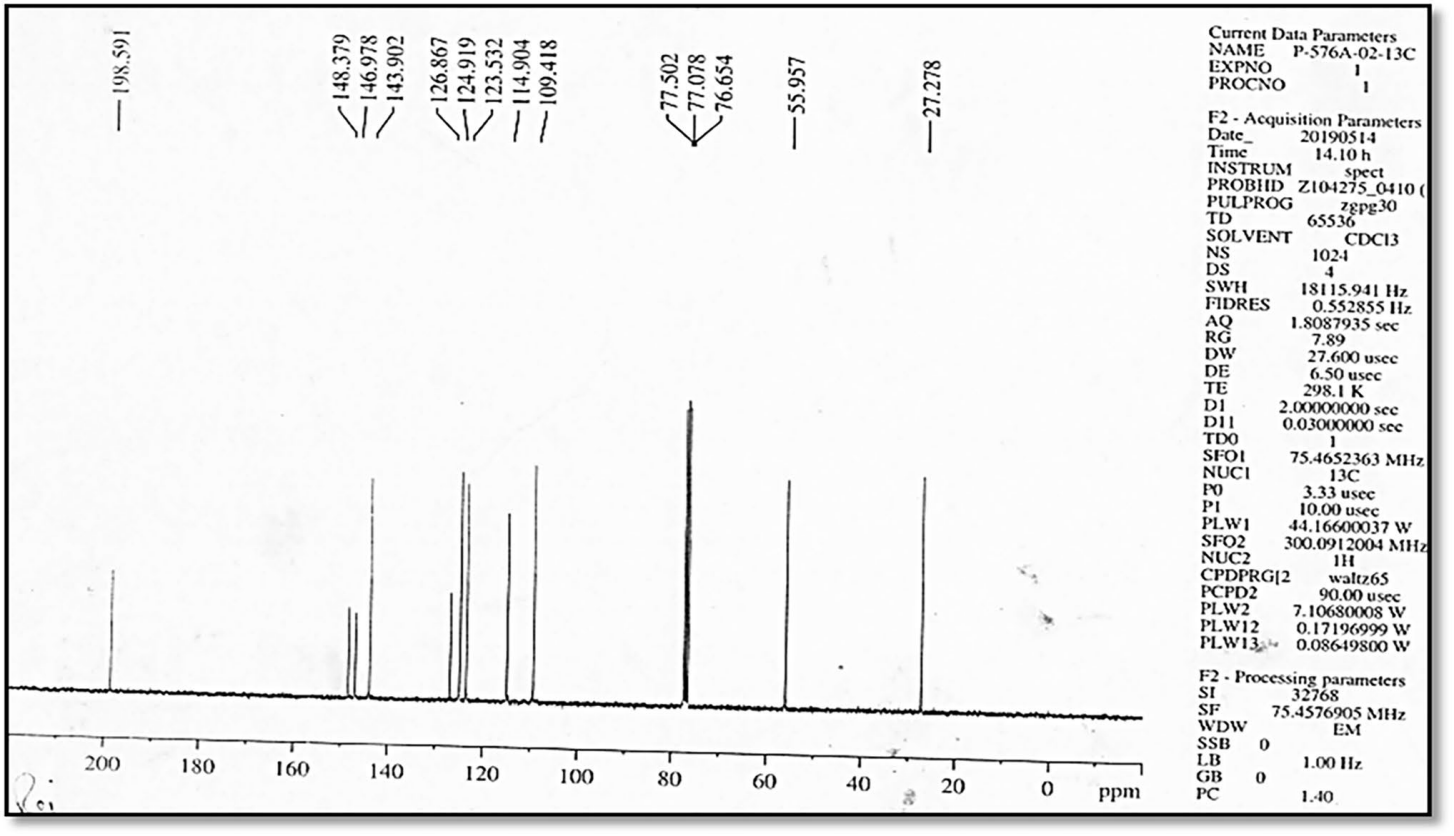
^13^C NMR of synthesized dehydrozingerone

**Fig. 4:**
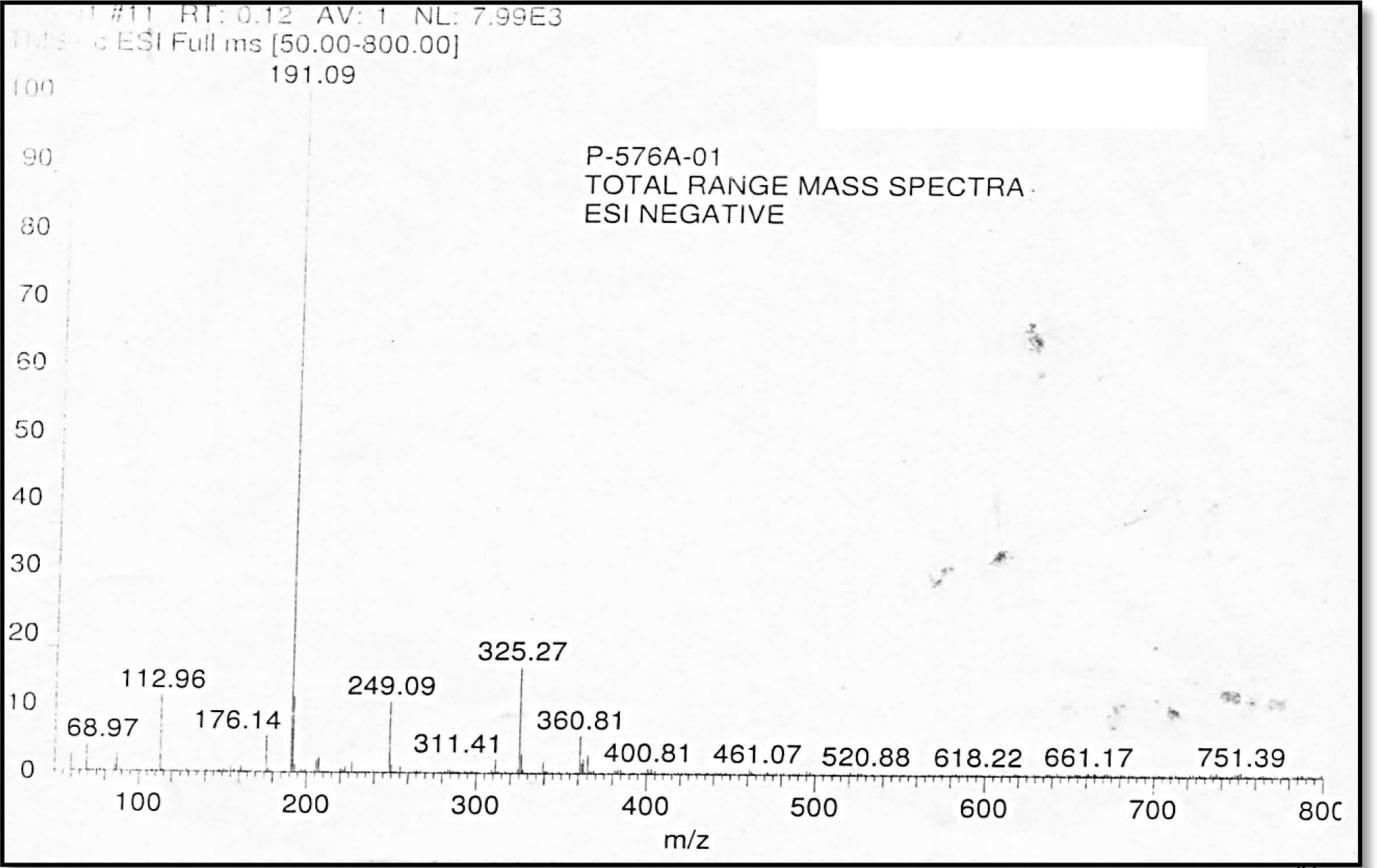
Mass spectra of synthesized dehydrozingerone. Mass spectra showed the parent ion peak at [M + 1] 191.09 m*/z* with 80% relative intensity consistent with the known molecular weight of dehydrozingerone which confirmed the product to be DHZ

The synthesis of DHZ was done using vanillin and acetone as starting materials. The final product obtained were shiny yellow crystals and the melting point was found to be 126 °C. IR (Fig. 1), ^1^H (Fig. 2), ^13^C NMR (Fig. 3), and mass spectra (Fig. 4) of the synthesized drug were obtained.

FT-IR spectra represented bands at 3290 (broad band), 3001, 2948.54, 2848.78, 1637, 1618, 1582, 1452, 1426, 1365, 1316, 1296, 1266, 1225, 1186, 1123, 1025, 1008, 981, 941, 823, 805, 759, 678, 606, 545, and 470 cm^−1^. The characteristic and diagnostic bands were visible at regions (3290–1582 cm^−1^). Aliphatic C–H stretching appeared at 2948 cm^−1^ and 2848 cm^−1^. Aromatic C=C stretching appeared at 1637 cm^−1^ and 1582 cm^−1^. Aryl C–H stretching bands were seen at 3001 cm^−1^. Stretching of the O–H bond was seen at 3290 cm^−1^. The bands of aryl alkyl ether have two characteristic peaks, the asymmetric C–O–C stretch was seen at 1266 cm^−1^ and a symmetric stretch at 1025 cm^−1^.

The ^1^H NMR spectrum showed the presence of CO–CH_3_ (2.3 δ), –OCH_3_ (3.9 δ), –OH (5.9 δ), =CH CO (doublet) (6.5–6.6 δ), Ar. CH= (doublet) (6.92–6.94 δ), m.2H. ArH (7.05–7.11 δ), d. 1H. ArH (7.42–7.47 δ), and CDCl_3_ (7.26). ^13^C NMR showed CH_3_ (27 δ), –OCH_3_ (55 δ), CDCL_3_ (77 δ), Ar–CH=CH (8 carbons) (109.148 δ), and C=O (198 δ). Mass spectra showed the parent ion peak at [M + 1] 191.09 m*/z* with 80% relative intensity consistent with the known molecular weight of dehydrozingerone (Jaganathan et al. 2018). All the above spectral analysis confirmed the product to be DHZ.

Dehydrozingerone solubility was determined in water and in phosphate buffer pH 7.4 and 5.6. DHZ was found to be more soluble in water when compared to phosphate buffer (Table 1).

**Table 1:** Solubility of dehydrozingerone (DHZ)

Water	1.20 ± 0.02 mg/mL
Phosphate buffer pH 7.4	0.035 ± 0.03 mg/mL
Phosphate buffer pH 5.6	0.016 ± 0.01 mg/mL

**Fig. 5:**
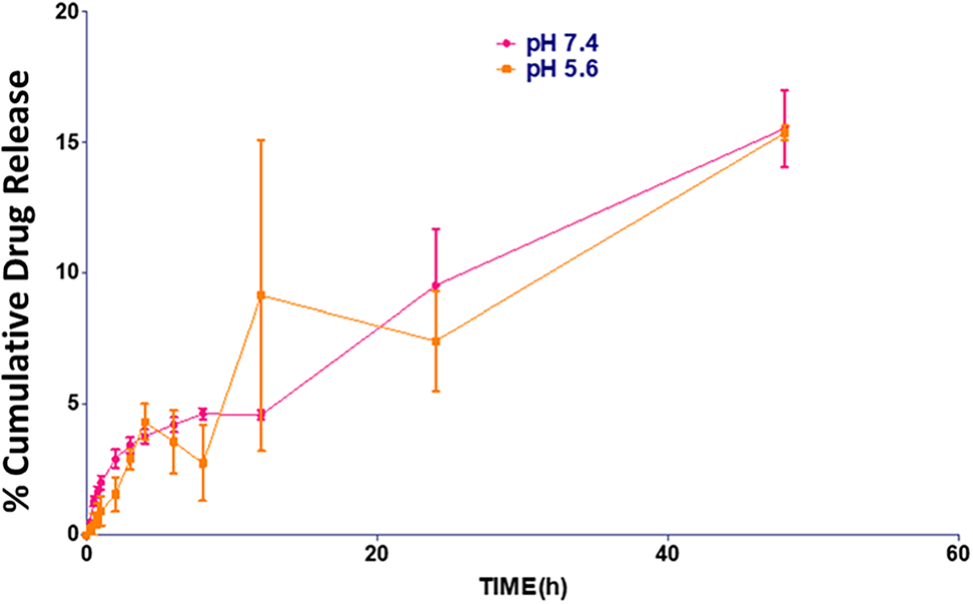
In vitro** cumulative drug release** percentage of DHZ gel formulation using phosphate-buffered saline pH 7.4 and pH 5.6

Among all the topical semisolid formulations, gel is preferred mostly due to its long duration on skin, viscosity, less irritation, ease of application and spreadability, better release characteristics, pH of the gel being 6, spreadability 40.22 mm^2^/g, viscosity 14.9 Pa s., and drug content 82% (Table 2). In vitro permeation studies were performed (Fig. 5); phosphate-buffered saline pH 7.4 and 5.6 was used for the in vitro studies of gel and the drug release kinetics is shown in Table 3.

**Table 2:** pH, spreadability, and drug content of DHZ gel

Evaluation parameters	Result
pH	6.530 ± 0992
Spread ability	40.2261 ± 343 (mm^2^/g)
Viscosity	14.9 Pa. s,
Drug content	82 ± 1.5%

**Table 3:** Drug release kinetics of DHZ gel

Kinetic parameter	Drug release media	
	7.4 pH	5.6 pH
Zero order (*R*^2^)	0.95	0.87
First order (*R*^2^)	0.96	0.88
Hixon Crowell (*R*^2^)	0.96	0.88
Higuchi (*R*^2^)	0.95	0.9
Korsmeyer–Peppas (*n*)	0.49	0.62

**Fig. 6:**
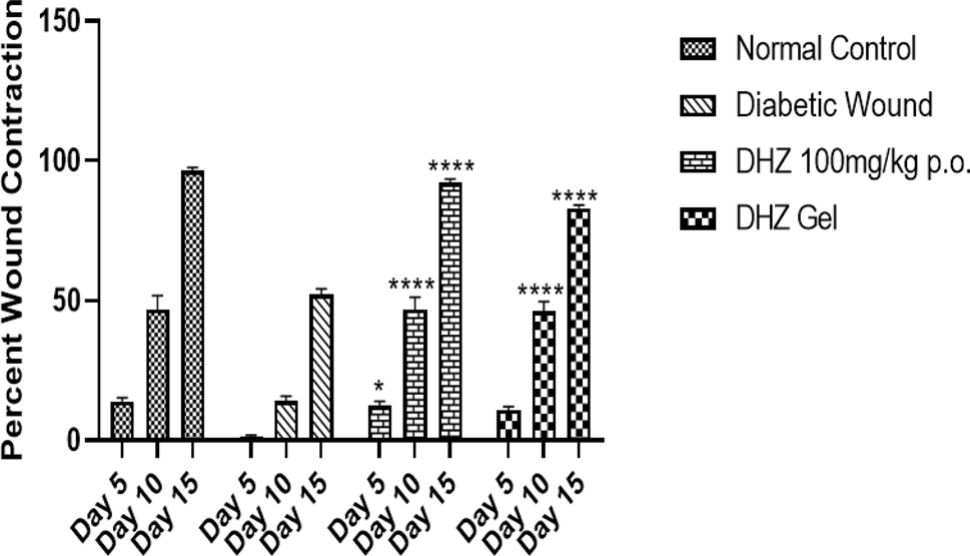
Effect of DHZ on the 5th, 10th, and 15th day of wound contraction. Data represented as mean ± SEM. **p < 0.001, ****p < 0.0001 when compared to the **excisional wound grou**p. Analysis was done using two-way ANOVA with Tukey’s post hoc test

Percentage wound contraction was measured using Image J software, and it was observed that the systemic DHZ (94%) (94.32 ± 1.44) and DHZ gel (81%) (81.58 ± 0.29) groups showed significant (*p* < 0.0001) improvement in wound closure on day 15 when compared to diabetic wounds (52%) (52.16 ± 2.09) (Fig. 6).

**Fig. 7:**
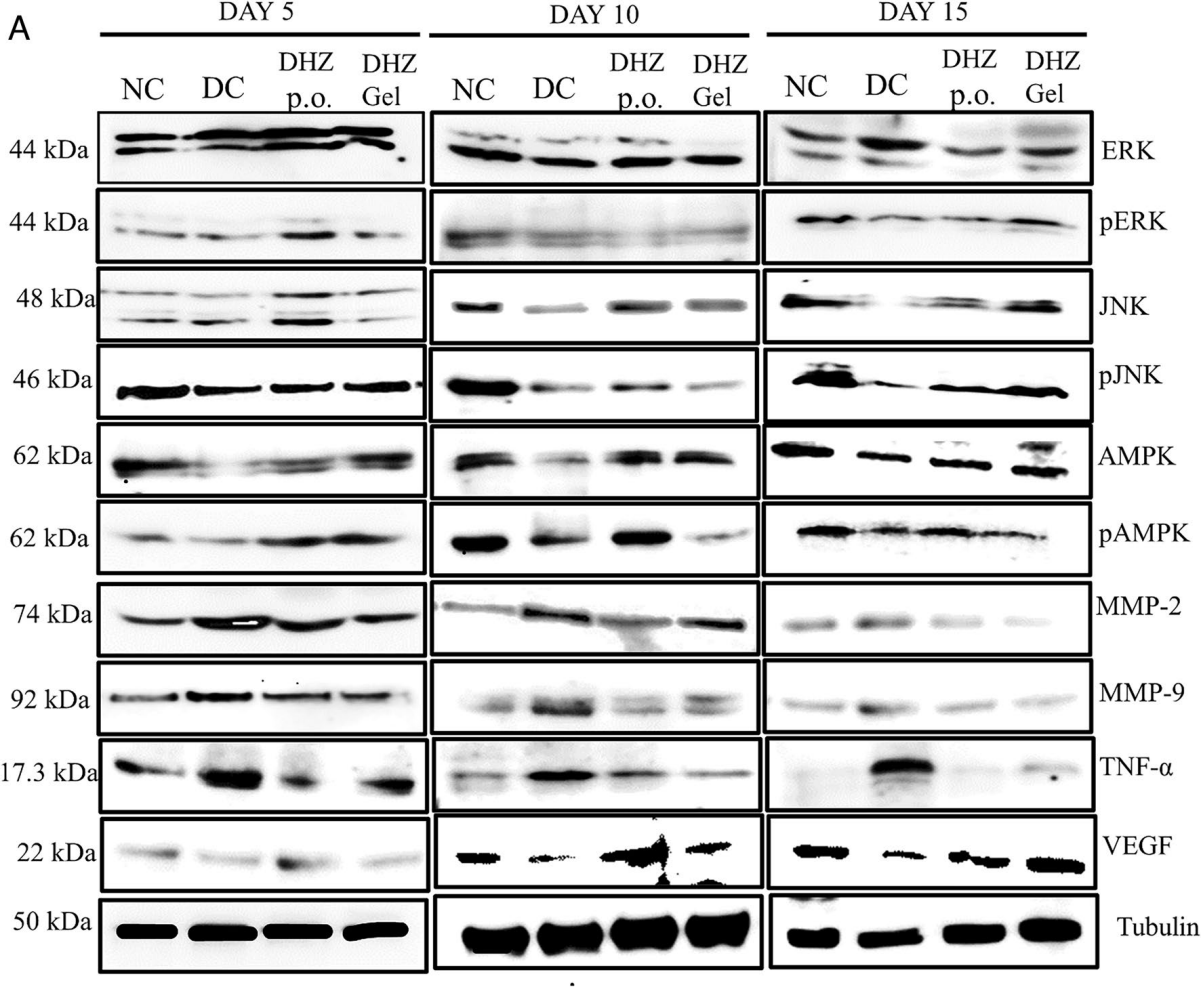

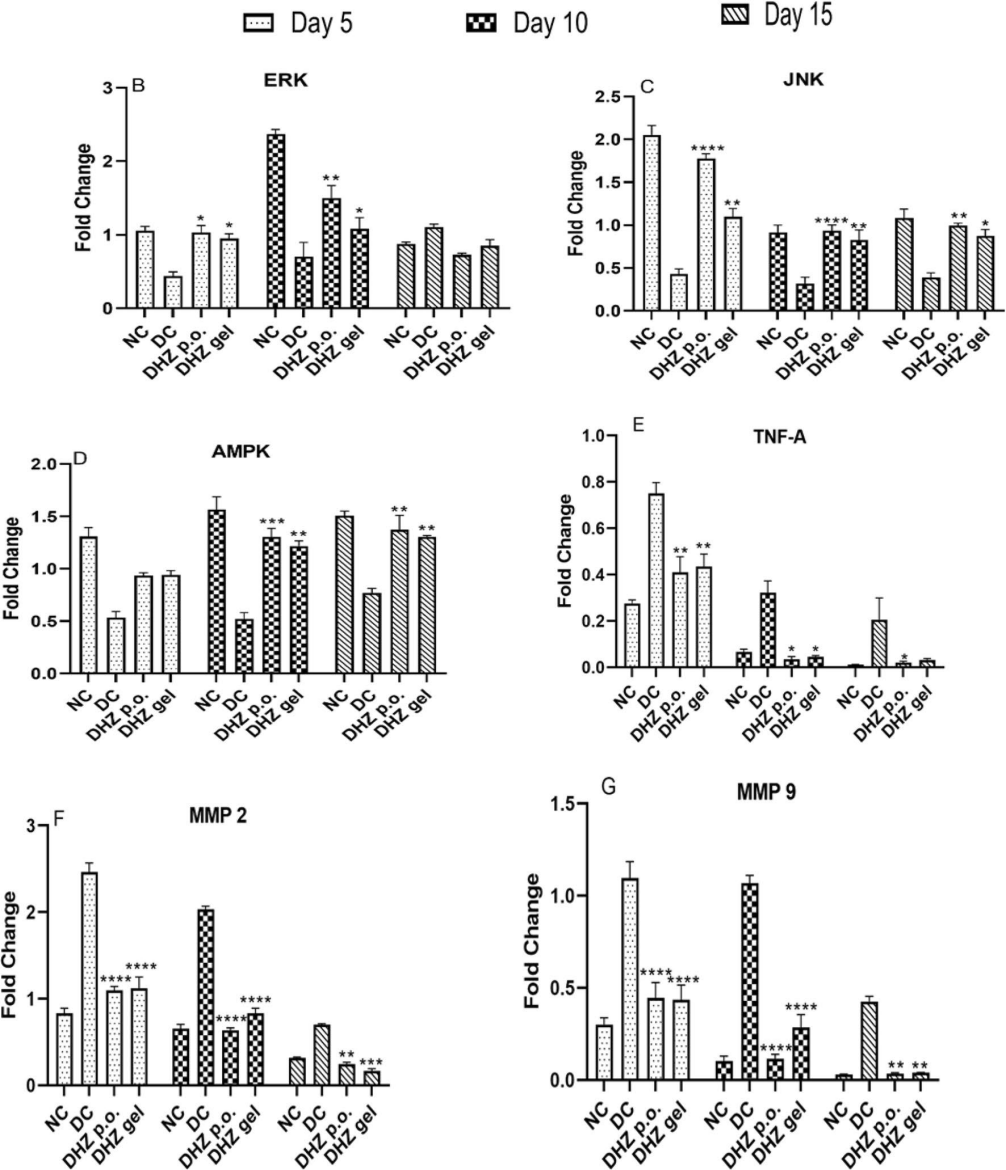
Effect of DHZ on ERK, JNK, AMPK, TNF-α, MMP-2, and MMP-9 on day 5, 10, and 15. **A** Representative images of blots. **B** p-ERK/ERK ratio, **C** p-JNK/JNK ratio, **D** p-AMPK/AMPK ratio, **E** TNF-α/α-tubulin ratio, **F** MMP-2/α-tubulin ratio, **G** MMP-9/α-tubulin ratio. Data represented as mean ± SEM. *p* < 0.00001, *p* < 0.0001, *p* < 0.001, *p* < 0.01. Analysis done using one-way ANOVA with Tukey’s post hoc test. Comparison for significance was observed between day 5 of treated vs day 5 of disease group, day 10 of treated group vs day 10 of disease group, and day 15 of treated group vs day 15 of disease group

Western blotting analysis was done and the ERK expression showed a gradual increase from day 5 to 10 in both the normal and treated groups (*p* < 0.001) (Fig. 7B). **In case of disease control**, significant increase was observed on day 15, which indicated that cell proliferation and differentiation were a part of MAPK signaling that occurred in the initial days of the normal and treated groups, resulting in early healing. On the contrary, cell proliferation and differentiation started at a slow phase in the disease group i.e., on day 15, resulting in delayed healing compared to the normal and treated groups. JNK expression showed a significant increase (*p* < 0.0001, *p* < 0.001, *p* < 0.1)) in the normal and treated groups from day 5 to 15 when compared to the disease group (Fig. 7C).

AMPK expression was non-significant in the treated groups on day 5, but the normal control group observed a gradual increase in the AMPK expression from day 5 to 15. Significant increase was observed in the treated groups (*p* < 0.0001, *p* < 0.001) on day 10 and 15 when compared to the disease control groups. Normal wounds showed increase in AMPK expression from day 5 onward, and hence quick healing was observed in normal wounds when compared to diabetic wounds (Fig. 7D). Gradual decrease in the TNF-α expression was observed in the normal and treated groups from day 5 to 15 when compared to the disease group (Fig. 7E).

A gradual decrease was observed in the expression of MMP 2 & 9 from day 5 to 15 in both normal and treated groups, which indicates that DHZ treatment decreased the MMP levels, thus, by creating a balanced production of extracellular matrix formation, healing the diabetic wound. The disease groups showed high expression of MMPs when compared to the normal and treated groups (Fig. 7F, G).

**Fig. 8:**
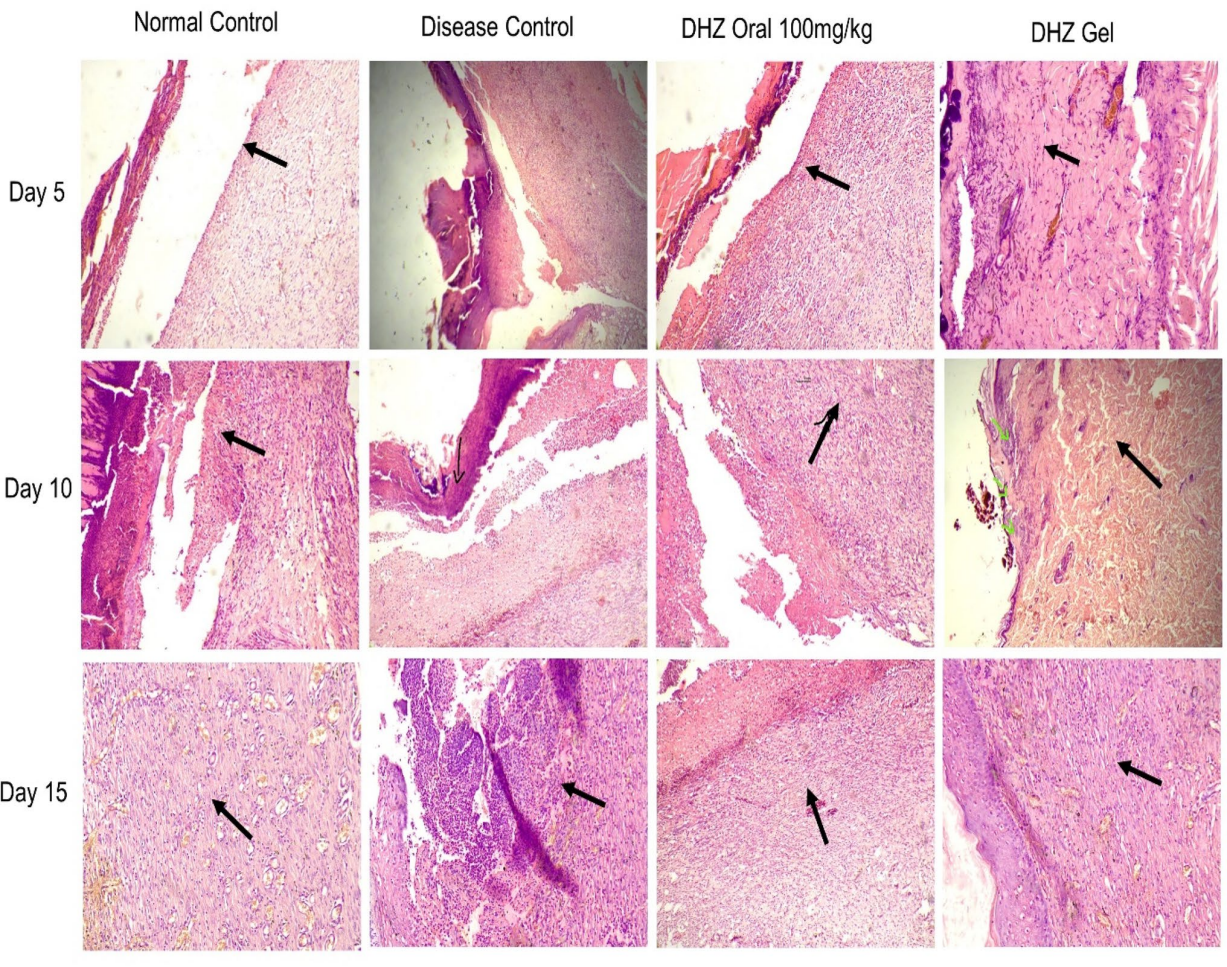
Effect of DHZ on histopathology of skin(H&E staining). Arrows indicate granulation tissue formation on day 5, 10, and 15 of wound healing along with well-developed epithelial layer on day 15 in both normal and treated groups. Optical zoom at 100×

A histopathological analysis of the normal control and treated groups showed the formation of granulation tissue and epithelial tissue earlier on day 5, when compared to the disease group. Ulcerative epithelium along with scab was observed in the disease and treated groups. When compared to disease group normal and treated groups exhibited reduced cell debris, abundance of neutrophils, extravasated RBC and fibrin. Granulation tissue along with proliferative fibroblasts, collagen, and many newly formed blood vessels were observed in the normal and treated groups on day 1 (Fig. 8).

**Fig. 9:**
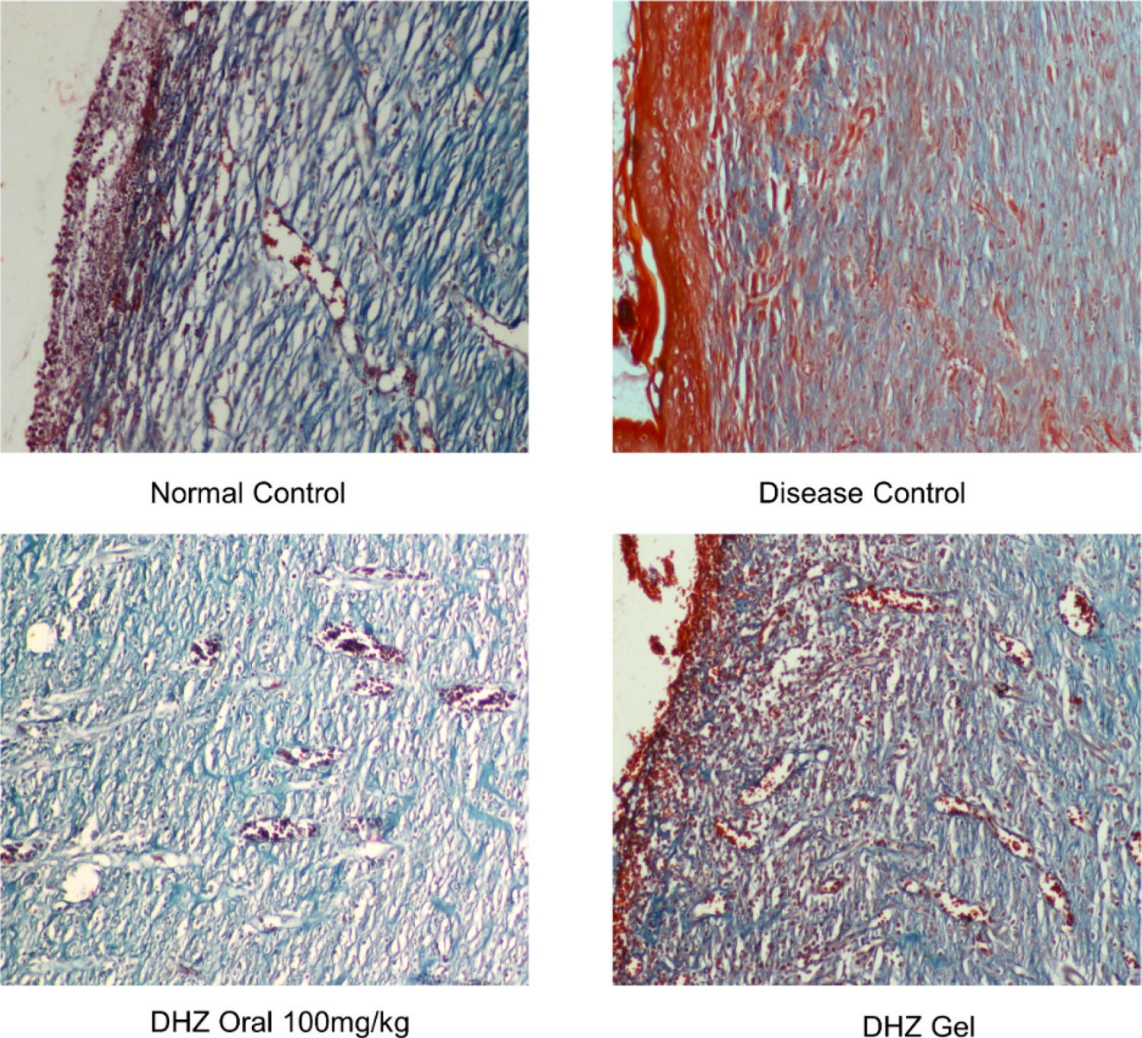
Effect of DHZ on collagen deposition (Masson trichrome staining) on day 15 of wound healing. Optical zoom at 100×

Masson’s trichrome staining was also performed to assess the collagen deposition at the end of day 15. Generally normal wound healing shows a regulated formation of collagen till healing itself and thus prevents scar formation. Treated groups showed a similar effect to the normal control group. Normal and treated groups showed more formation of collagen on day 15 when compared to the disease group (Fig. 9).

**Fig. 10:**
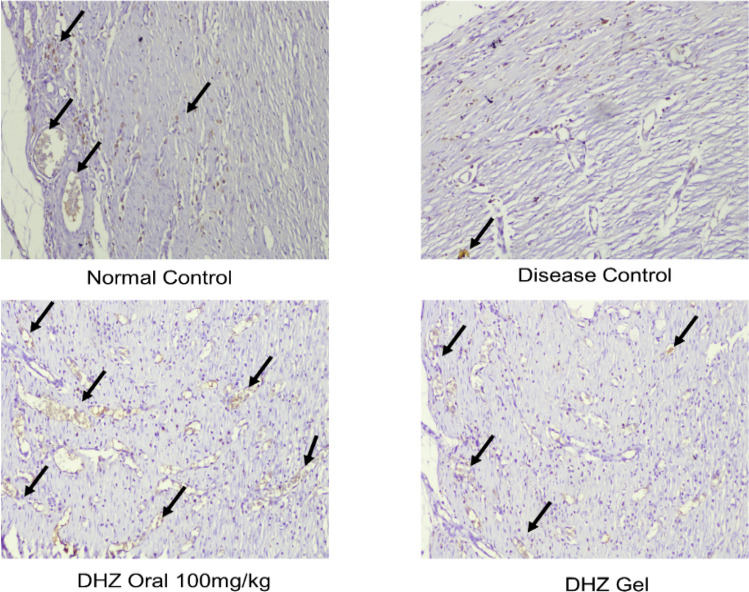
Effect of DHZ on immunostaining using CD-31 marker for angiogenesis on day 15 of wound healing. Arrows indicate angiogenesis. Optical zoom at 100×

CD31 immunostaining was investigated on day 15 to confirm neo vessel formation. This protein is a highly specific marker for endothelial cells. CD-31, in other words, is a cluster of differentiation-31 or PECAM-1 (platelet endothelial cell adhesion molecule), which is a marker for angiogenesis and expressed on the endothelial cell surfaces (Bitto et al. 2013). Positive staining was observed in the treated wounds (Fig. 10). CD-31 staining was markedly reduced in the disease control group (8 ± 2). Administration of oral DHZ (35 ± 4) and application of topical DHZ gel (28 ± 2) augmented immunostaining of CD-31 in rats (Fig. 10). In western blot data, treated groups showed VEGF expression along with normal control group, whereas the expression was less in the disease group which can be correlated with CD-31 immunostaining.

## Discussion

In this study, dehydrozingerone has shown beneficial effects in accelerating healing in diabetic rats, which was observed with shrinkage of wounds. Oral administration and topical DHZ gel application favored the healing process in the excision wound model of diabetic rats compared to normal control. The percentage decrease in the wound surface area was found to increase in a time-dependent manner in both oral and topical gel groups. The rate of wound contraction was significantly higher on day 15 in systemic DHZ (94%) and DHZ gel (81%) when compared to disease control (52%). Contraction of wounds is a healing response that functions by decreasing the wound area, thus reducing the damage of tissue that requires repair. The repair of wounds involves myofibroblasts, developed from the alpha-smooth muscle actin (α-SMA) gene, which in turn is activated by fibroblasts (Rao et al. 2011; Aloysius Ajoy et al. 2022). These fibroblasts move into the wound tissue, join together with the wound, and empower wound closure (Keni et al. 2020). This process occurs only when collagen, an extracellular matrix, has been deposited (Brauer et al. 2019). Collagen and extracellular matrix (ECM) deposits enable the tissue cells to form attachments, change, and eventually settle down permanently in the wound’s healing area (Keni et al. 2020). Using Masson trichrome staining, we confirmed the collagen deposition (Fig. 9), and through H&E staining, we confirmed the formation of granulation tissue (Fig. 8).

Collagen 1 is a protein molecule that fragments naturally during the wound-healing process. This fragmentation of collagen 1 involves matrix metalloproteinases, which play a major role in the healing and formation of scars (León-López et al. 2019). Diabetic wounds have deficient wound-healing properties and accompany disturbed collagen metabolism at the site of the wounds. The prolonged formation of collagen may lead to abnormal scar formation on the healed wound (Goulding 2015). Epithelium formation, fibroblast formation, collagen formation, and angiogenesis are considered hallmarks of wound healing, which have been observed in the normal and treated groups (Begum et al. 2022). Angiogenesis was confirmed through immunostaining using the CD-31 marker (Fig. 10).

The mitogen-activated protein kinase (MAPK) signaling pathway includes kinases like ERK and JNK, which regulate the cellular process, viz., differentiation, proliferation, and apoptosis (Guo et al. 2020). The expression of ERK and JNK was found to be increasing toward day 15, which is almost similar to that of normal healing process, as seen in western blot analysis (Fig. 7B, C). AMPK activation helps to alleviate tissue inflammation and accelerate re-epithelialization, thus helping the diabetic wound to heal (Lin et al. 2014). From the study results, it was hypothesized that AMPK was activated significantly from day 10 onward in the treated groups, thus helping to re-epithelialize wound tissue (Fig. 7D). Prolonged levels of TNF-α lead to impaired proliferation of fibroblasts, especially in diabetic wounds (Xu et al. 2013). Research studies have reported that TNF-α promotes apoptosis of keratinocytes, endothelial cells, and fibroblasts in vitro (Petrache et al. 2000; Xu et al. 2013). Diabetic wounds involve impaired migration of keratinocytes and fibroblasts (Werner et al. 2007) due to increased TNF-α levels, which inhibit cell migration (Corredor et al. 2003). Moreover, the cellular ability to respond to insulin at the wound site gets reduced in diabetic wounds, resulting in insulin insensitivity. Decreased levels of TNF-α in the diabetic wound enhance wound closure and angiogenesis (Fig. 7E) (Xu et al. 2013). MMPs are produced from various cell types like keratinocytes, fibroblasts, and inflammatory cells during various phases of wound healing. This process is regulated in a controlled and coordinated way (Tombulturk et al. 2019). Both MMP 2 and 9 are involved in the degradation of collagen through cell migration and granulation tissue formation (Tombulturk et al. 2019). MMPs are involved in the degradation of the components of the extracellular matrix. In diabetic wounds, there will be overexpression of MMPs, resulting in increased degradation of extracellular matrix components, making wound healing difficult (Muller et al. 2008). Studies have revealed the connection between diabetes and overexpression of MMPs in wound tissue (Shiau et al. 2006; Gharagozlian et al. 2009). However, the mechanism of deranged healing is unclear regarding the link between MMPs and diabetes. There is an explanation that it is due to increased monocyte-macrophage activation due to chronic circulating blood glucose levels, which induces cytokines, resulting in the overexpression of MMPs (Tombulturk et al. 2019). The western blot analysis (Fig. 7F, G) shows that the levels of MMP 2 and 9 were significantly decreased in both the treatment groups compared to disease control.

From the study results, it can be concluded that DHZ in both systemic and topical gel formulations can improve the rate of diabetic wound healing. It accelerates the deposition of collagen and shrinkage of the wound, thus making the closure of the wound faster when compared to diabetic non-healing wounds.

## Conclusion

The study results revealed that dehydrozingerone (both topical and systemic) showed a positive effect and can regulate the hallmarks involved in wound healing. The percentage decrease in the wound surface area was found to increase in a time-dependent manner in both the oral and topical gel groups. It showed a significant response as in normal wounds, i.e., some hallmarks started expressing from day 5 onward which indicates that DHZ can repair diabetic wounds at a faster rate by effectively showing anti-inflammatory effect by decreasing TNF-α and MMPs, formation of granulation tissue and collagen, activating MAPK signaling, and promoting angiogenesis.

## Data Availability

Not applicable.

## Abbreviations

ROS: Reactive oxygen species
DHZ: Dehydrozingerone
IR: Infrared spectroscopy
NMR: Nuclear magnetic resonance
ERK: Extracellular signal-regulated kinase
p-ERK: Phospho extracellular signal-regulated kinase
JNK: c-Jun *N*-terminal kinases
p-JNK: Phospho c-Jun *N*-terminal kinases
AMPK: 5′ Adenosine monophosphate-activated protein kinase
pAMPK: Phospho 5′ adenosine monophosphate-activated protein kinase
VEGF: Vascular endothelial growth factor
MMP-2: Matrix metalloproteinase-2
MMP-9: Matrix metalloproteinase-9
TNF-α: Tumour necrosis factor
SMA: Smooth muscle actin
COL: Collagen
CD31: Cluster of differentiation
H&E: Hematoxylin and eosin staining

## Declarations

### Patent

Dehydrozingerone gel formulation, and a method for preparing the same'' has been filed with the Indian Patent Office bearing the application number 202241050289 on 02 September 2022. Current status: file under RFE process.
